# Global trends and knowledge structure of cardiovascular magnetocardiography research: a bibliometric analysis

**DOI:** 10.3389/fmed.2026.1960096

**Published:** 2026-10-02

**Authors:** Yaxing He, Xu Zhang, Xiaole Han, Zifeng Zhang, Shiqiang Zheng, Kangqi Tian, Min Xiang

**Affiliations:** 1 School of Instrumentation and Optoelectronic Engineering, Beihang University, Beijing, China; 2 Zhejiang Key Laboratory of Zero Magnetic Medicine, Hangzhou, China; 3 Zhejiang Provincial Key Laboratory of Ultra-Weak Magnetic-Field Space and Applied Technology, Hangzhou, China; 4 Key Laboratory of Ultra-Weak Magnetic Field Measurement Technology (Beihang University), Ministry of Education, Beijing, China; 5 Hangzhou Institute of Extremely-Weak Magnetic Field Major National Science and Technology Infrastructure, Hangzhou, China

**Keywords:** bibliometrics, cardiovascular diseases, global trend, literature metrics analysis, magnetocardiography

## Abstract

**Background:**

Cardiovascular diseases remain a leading global health threat. Recent advances in room-temperature sensing and artificial intelligence (AI) have renewed research interest in the clinical translation of magnetocardiography (MCG) for cardiovascular diagnosis, yet the global research evolution of MCG has yet to be quantitatively characterized.

**Methods:**

Literature from the Web of Science Core Collection and Scopus (2000–2026) was analyzed using CiteSpace, VOSviewer, and R to evaluate publication trends, knowledge networks, and evolving frontiers.

**Results:**

A total of 631 WoSCC and 755 Scopus articles were included. The publication trajectory exhibits a prolonged plateau followed by substantial recent growth. Germany, the United States, and China are the most prolific contributors. Keyword analysis revealed a diversification of technological and analytical themes. While early literature was strongly associated with superconducting quantum interference devices-based MCG, recent research has increasingly highlighted optically pumped magnetometers, magnetic-field control, sensor arrays, magnetic noise suppression, and AI-based signal analysis. The application landscape has broadened from physiological validation to coronary artery disease diagnosis, arrhythmia assessment, and fetal electrophysiology.

**Conclusion:**

Collectively, these findings suggest that the evolution of cardiovascular MCG reflects the parallel development and interaction of sensor technologies, AI-based analysis, and emerging clinical applications. Future research should prioritize standardized protocols, scalable and robust acquisition systems, and explainable AI models to accelerate clinical implementation.

## 1 Introduction

Cardiovascular diseases impose a severe global health burden, representing the leading cause of mortality worldwide (1). While traditional diagnostic tools such as electrocardiography (ECG) are ubiquitous, they are inherently limited by the volume conduction effect, where electrical signals are attenuated and distorted by tissues with varying conductivities (2). In contrast, magnetocardiography (MCG) has emerged as a promising, non-invasive modality. By measuring the ultra-weak magnetic fields generated by cardiac electrophysiological activity, MCG is less affected by tissue distortions, thereby offering improved spatial resolution. One study has demonstrated promising sensitivity in identifying ischemic conditions when compared to standard ECG (3).

The field of MCG is intrinsically interdisciplinary, situated at the nexus of cardiovascular medicine, quantum sensing, and instrumentation engineering. Since 2000, MCG instrumentation has evolved through the parallel development and optimization of mature superconducting quantum interference device (SQUID) systems and emerging room-temperature optically pumped magnetometers (OPMs) (4). The continuous maturation of these sensor technologies, coupled with the growing clinical demand for precise and non-invasive cardiovascular diagnostics, is continuously reshaping the research landscape of this field. While several reviews have been published, they remain largely qualitative (2, 4, 5). A systematic and quantitative bibliometric analysis is thus necessary to accurately map these multidisciplinary shifts.

To bridge these gaps, this study employs bibliometric methods and science mapping techniques to systematically analyze global research outputs on cardiovascular-related MCG over the past 26 years. Specifically, this study aims to address three primary research questions: (1) What is the overall developmental landscape and global trajectory of MCG research? (2) Which countries, institutions, journals and authors have shaped the knowledge structure? (3) How have the intellectual structure and research frontiers evolved alongside recent technological advancements? By visualizing the evolutionary pathways, identifying emerging hotspots, and mapping the intellectual structure, this study aims to provide a detailed landscape of the field’s developmental dynamics and offer objective insights into future clinical translation and technological trends.

## 2 Methods

### 2.1 Data sources and search strategy

The literature search was conducted on July 8, 2026, across the Web of Science Core Collection (WoSCC) and the Scopus database. WoSCC, specifically the Science Citation Index Expanded, was selected as the primary database due to its standardized citation records and high compatibility with knowledge mapping software (6, 7). Scopus was utilized as an independent dataset to evaluate the consistency of the findings, specifically regarding temporal publication trends and journal distribution.

Given the interdisciplinary nature of MCG, the search strategy was designed to broadly capture cardiovascular-related MCG research. The retrieval relied on a combination of two primary conceptual domains: magnetic sensing technologies and clinical cardiovascular applications. Key search terms for the sensing domain included “MCG,” “SQUID,” and “OPM”. For the clinical domain, terms such as “cardiovascular,” “coronary artery disease (CAD),” “ischemia,” and “arrhythmia” were utilized. These terms were searched within the Topic (TS) field in WoSCC and the TITLE-ABS-KEY field in Scopus. The exact Boolean search strings for both databases are provided in the Supplementary Material.

The retrieval timeframe spanned from January 1, 2000, to July 8, 2026. Although pioneering MCG studies appeared in the 1960s (8), this analysis focused on publications from 2000 onwards for two reasons. First, electronic indexing became more standardized during this period, improving the reliability of the bibliometric data (7). Second, the post-2000 era represents a critical translational stage for the field. This period is characterized by parallel advancements in sensor instrumentation, sophisticated signal analysis, and expanded clinical validations (4).

### 2.2 Inclusion and exclusion criteria

Following the PRISMA guidelines (9), the initial search yielded 1,951 records in WoSCC and 4,708 in Scopus. Filters were applied to restrict the document types to Articles and Review Articles published in English. Subsequent manual screening of titles, abstracts, and full texts was conducted to exclude irrelevant studies, non-journal publications, and conference records. Consequently, a final dataset comprising 631 articles from WoSCC and 755 articles from Scopus was established. The two databases shared 548 records, accounting for 65.4% of the merged dataset (*n* = 838). The detailed literature screening and selection process is illustrated in Figure 1. Two investigators independently screened titles, abstracts, and full texts, and discrepancies were resolved through discussion.

**Figure 1:**
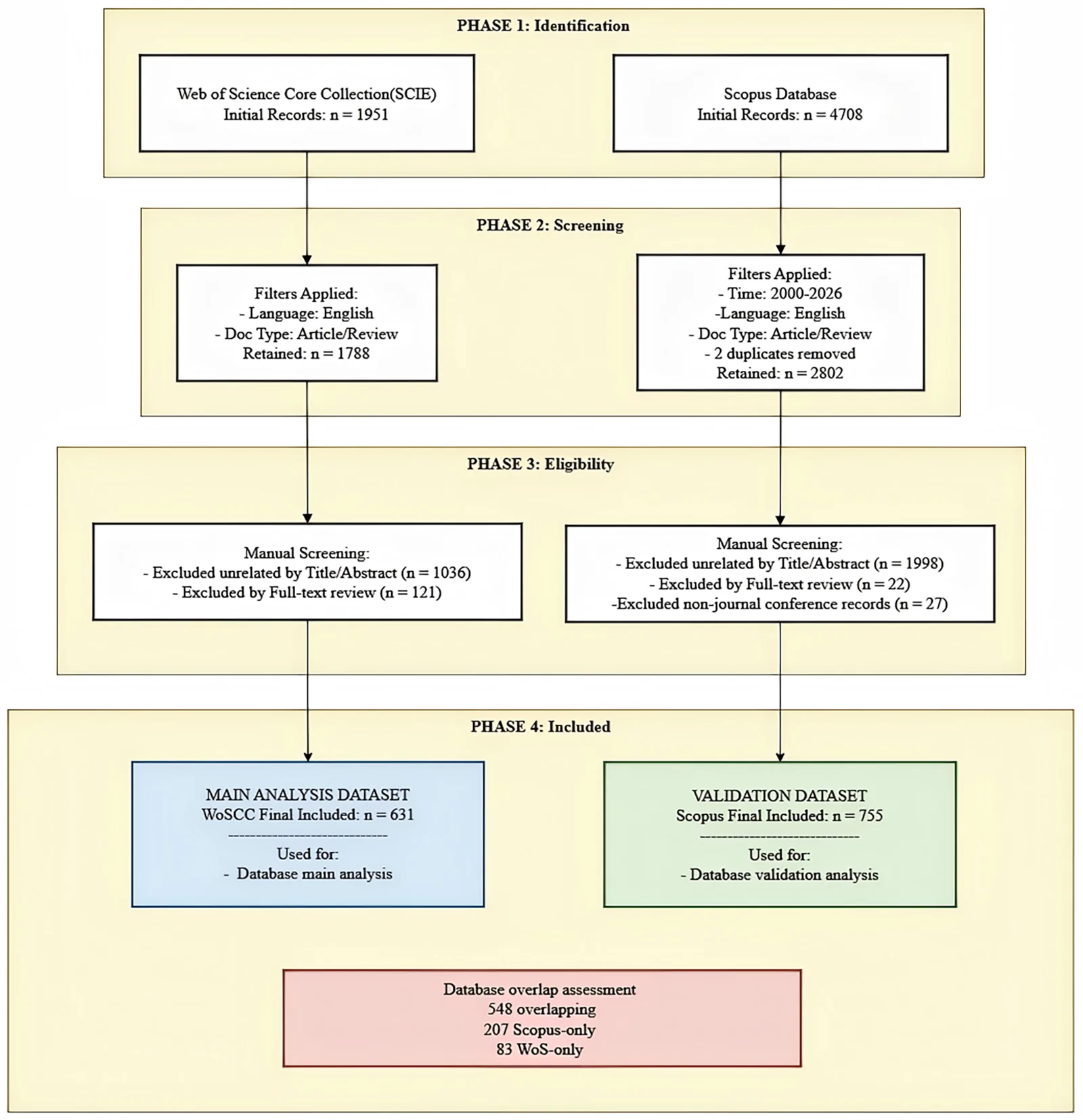
PRISMA flow diagram for the literature search and selection process.

### 2.3 Bibliometric analysis

Bibliometrics, a quantitative analytical method rooted in mathematics and statistics, offers the capability to evaluate research performance and visually elucidate a field’s collaborative networks, thematic evolution, and foundational knowledge through knowledge map visualization (10). By transforming qualitative literature into quantitative metrics, it provides an objective, macro-level perspective on global research trends, research hotspots, and interdisciplinary knowledge flows (11).

Prior to data visualization, a rigorous data cleaning process was conducted to prevent data dispersion and ensure analytical accuracy. This procedure included resolving author name ambiguities and standardizing geographical regions, such as unifying England, Scotland, Wales, and Northern Ireland under the United Kingdom. Keyword variants, abbreviations, and plural forms were harmonized into uniform terms, ensuring that variations like magnetocardiogram, MCG, and magnetocardiography were consistently indexed as magnetocardiography.

This study primarily utilized VOSviewer (version 1.6.21), CiteSpace (version 6.4.R1), SCImago Graphica (version 1.0.55), R software (version 4.5.1), and Microsoft Excel (version 2016) for data processing and graph construction. Specifically, VOSviewer was employed to construct and visualize co-occurrence, co-authorship, citation, and co-citation networks. In VOSviewer maps, node size represents item frequency or weight, link thickness reflects association strength, and color indicates cluster membership or the average publication year, depending on the specific map type (12). CiteSpace was utilized to identify prominent keywords, generate timeline views, dual-map overlay of journals and perform citation burst detection to track the evolution of research frontiers (11, 13). In the CiteSpace burst maps, burst strength and red bars indicate the intensity and active period of sudden increases in academic attention. SCImago Graphica was used to create a country/region co-authorship overlay map incorporating a global geographical map, as well as a collaboration ring chart for top-producing institutions (14). Microsoft Excel was primarily utilized for organizing, filtering, and integrating literature data, and for charting annual publication trends. Finally, R software was used to generate Venn diagrams and side-by-side bar charts to compare the journal coverage and publication distribution between the WoSCC and Scopus databases. Detailed parameter configurations for all VOSviewer and CiteSpace visualizations are provided in the Supplementary Material.

### 2.4 Technology-stratified review of representative studies

To bridge the bibliometric findings with their clinical implications, representative clinical and translational studies discussed in the manuscript were manually reviewed. Data were extracted regarding sensor technology, sensor configuration, magnetic environment, analysis method, clinical reference standard, and evidence level. These studies were selected to illustrate the field’s technological evolution rather than to serve as an independent systematic meta-analysis. Where specific methodological information was not explicitly reported in the source publication, it was recorded as not reported.

Based on the study objectives, the bibliometric analyses were structured into seven sequential aspects: (1) publication and citation trends; (2) collaboration networks across countries, regions, and organizations; (3) author productivity and co-authorship networks; (4) journal distribution and co-occurrence; (5) knowledge mapping and topic evolution; (6) intellectual foundations via co-citation analysis; and (7) methodological validation using the Scopus database.

## 3 Results

### 3.1 Publication and citation trends

The primary analysis of the 631 WoSCC articles revealed contributions from 2,145 authors across 544 institutions in 36 countries/regions. The literature was published across 183 journals, drawing upon 10,950 references from 3,086 source journals. As illustrated in Figure 2, the annual publication output (indicated by the blue bars) exhibits a multiphasic pattern rather than a consistent upward trajectory. Following a period of moderate activity in the early 2000s, the publication volume experienced a prolonged phase of fluctuation and relative stagnation, reaching its lowest point in 2021. However, the field witnessed a resurgence thereafter, culminating in a historical peak of over 60 publications in 2025. Concurrently, the annual citation count (indicated by the orange line) illustrates a steady, continuous accumulation over the past 26 years, followed by a sharp acceleration post-2022. Mirroring the publication surge, the citations surged to a maximum of over 1,600 in 2025.

**Figure 2:**
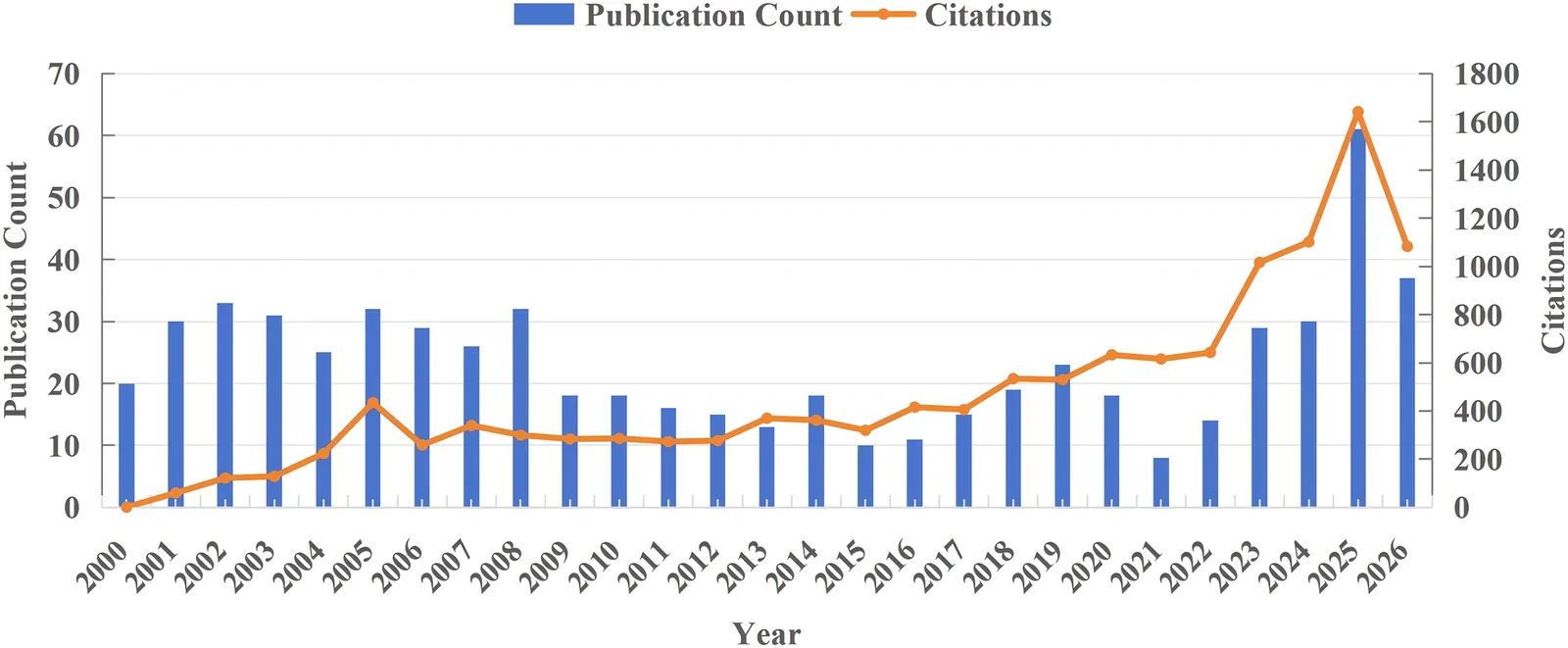
Annual publication output and citation trends based on the WoSCC database. The data for 2026 represents a partial year up to the search date.

### 3.2 Countries/regions and organizations

An analysis of national contributions (Table 1) indicates that Germany leads in total publication volume (158 documents), followed closely by China (154) and the USA (118). However, in terms of overall academic impact, Germany (3,657) and the USA (3,179) exhibit the highest total citations, significantly outperforming China (1,838). Interestingly, when evaluating the average citations per publication, the Netherlands (45.82) and the United Kingdom (31.13) rank at the forefront despite their relatively lower publication outputs.

**Table 1:** Ranking of the top 10 countries/regions contributing to the literature (Source: VOSviewer).

Rank	Country	Publications	Citations	Average citation/publication
1	Germany	158	3,657	23.15
2	China	154	1,838	11.94
3	USA	118	3,179	26.94
4	Japan	93	1,495	16.08
5	Italy	53	888	16.75
6	South Korea	34	529	15.56
7	Finland	33	747	22.64
8	United Kingdom	23	716	31.13
9	India	17	101	5.94
10	Netherlands	11	504	45.82

The geographical collaboration network (Figure 3A) further visualizes the global research landscape. In this map, the size of the nodes corresponds to a country’s publication volume, while the color intensity of the connecting lines (links) reflects the strength of international cooperation. Visual inspection reveals a hub-centric collaboration pattern rather than a dense, uniform global web. Specifically, Germany and the USA act as major international hubs, maintaining cooperative ties with numerous countries across Europe and beyond. In contrast, while China displays a prominent node indicative of its high research output, its external connecting lines are relatively faint. This observation, alongside the internal cohesion observed in the institutional collaboration ring (Figure 3B), suggests that China’s substantial publication volume is characterized by a limited international collaboration intensity, with research efforts appearing more concentrated within domestic cooperative consortiums.

**Figure 3:**
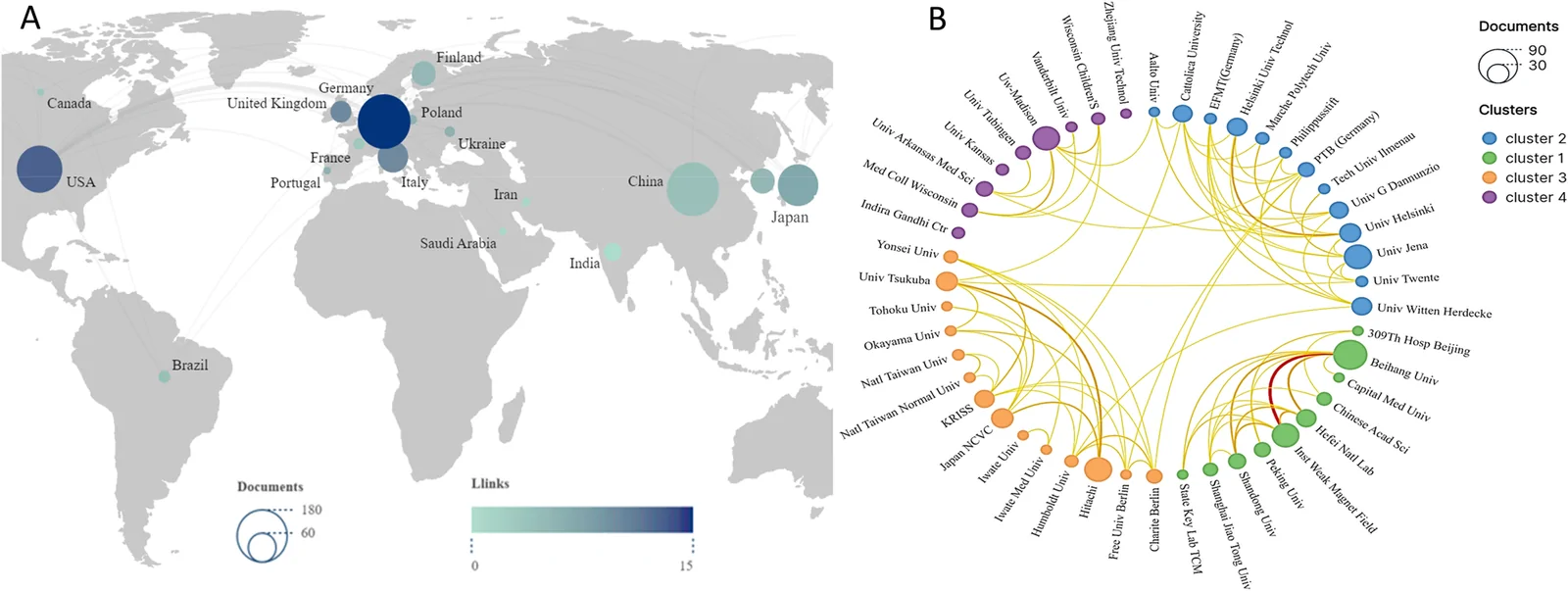
Regional and institutional collaboration networks. **(A)** Global collaboration map. **(B)** Collaboration ring map of the top 46 institutions. (Generated using VOSviewer and SCImago Graphica).

At the institutional level (Table 2), Beihang University ranks first in publication volume (64 documents), followed by the University of Jena (44), Hitachi (43), and the University of Wisconsin-Madison (UW-Madison, 42). The institutional collaboration ring (Figure 3B) delineates a pronounced regional clustering, which can be stratified into four major collaborative networks. Specifically, the blue cluster (cluster 2) forms a European precision measurement camp, encompassing key institutions such as the University of Jena (Germany), Cattolica University (Italy), and the University of Helsinki alongside the Helsinki University of Technology (Finland). The green cluster (cluster 1) represents a Chinese industry-academia-research consortium centered around Beihang University and the Institute of Extremely-Weak Magnetic Field Infrastructure, exhibiting strong internal cohesion. The orange cluster (cluster 3) is largely dominated by East Asian research powerhouses, including the University of Tsukuba, Hitachi Ltd., Japan’s National Cerebral and Cardiovascular Center (NCVC), and the Korea Research Institute of Standards and Science (KRISS). Meanwhile, the purple cluster (cluster 4) is predominantly a North American network dominated by UW-Madison and its affiliated clinical centers.

**Table 2:** Ranking of the top 10 organizations contributing to the literature (Source: VOSviewer).

Rank	Organization	Publications	Citations	Average citation/publication
1	Beihang University	64	712	11.13
2	University of Jena	44	1,081	24.57
3	Hitachi, Ltd.	43	740	17.21
4	University of Wisconsin–Madison	42	1,175	27.98
5	Institute of Extremely-Weak Magnetic Field Infrastructure	42	227	5.40
6	National Cerebral and Cardiovascular Center (Japan)	28	411	14.68
7	University of Tsukuba	27	642	23.78
8	University of Helsinki	27	596	22.07
9	University of Witten/Herdecke	24	601	25.04
10	Helsinki University of Technology	24	477	19.88

The “Institute of Extremely-Weak Magnetic Field Infrastructure” listed refers to its full official designation: Hangzhou Institute of Extremely-Weak Magnetic Field Major National Science and Technology Infrastructure.

### 3.3 Authors and co-authorship networks

To explore the distribution characteristics of research forces in this field, classic bibliometric laws were introduced. First, according to Price’s Law, the minimum publication threshold for core authors is calculated as $m=0.749\times \sqrt{N_{\max}}$, where $N_{\max}=41$ is the maximum number of publications by a single author in this field (15). Statistics show that there are 144 core authors with 5 or more publications (6.71% of total authors), contributing 1,329 signatures (32.86% of total signatures). This proportion is lower than the theoretical expectation of 50% proposed by Price’s Law, indicating that a highly consolidated core research community has not yet fully formed in this field.

Secondly, the publication distribution in this dataset conforms to the long-tail characteristics of Lotka’s Law (16). Among the 2,145 authors, 1,524 authors published only a single paper, accounting for 71.05%. This suggests a highly diversified scientific collaboration network, characterized by numerous one-time collaborators and peripheral explorers entering the interdisciplinary MCG space.

To systematically evaluate the leading contributors, a stricter threshold was applied. Authors were initially filtered by a high-yield criterion (≥20 publications), followed by the selection of the top 10 individuals based on their average citations per publication. The detailed productivity and academic impact of this subset are presented in Table 3. A. Kandori ranks first in publication volume, while J.F. Strasburger commands the highest total citations.

**Table 3:** Top 10 leading authors and their academic impact in MCG research.

Rank	Author	Publications	Citations	Average citation/publication
1	Strasburger, Janette F.	25	1,751	70.04
2	Wakai, Ronald T.	36	1,064	29.56
3	Van Leeuwen, Peter	26	671	25.81
4	Toivonen, Lauri	20	431	21.55
5	Brisinda, Donatella	23	459	19.96
6	Kandori, Akihiko	41	751	18.32
7	Tsukada, Keiji	32	575	17.97
8	Fenici, Riccardo	23	409	17.78
9	Haueisen, Jens	27	465	17.22
10	Miyashita, Tsuyoshi	30	466	15.53

Authors were initially filtered by a minimum of 20 publications and subsequently ranked by their average citations per publication.

While Table 3 highlights the individual impact of the leading researchers, understanding the collaborative dynamics and historical trajectory of the field requires mapping the broader core community. Therefore, a time-overlay co-occurrence network was constructed based on the core authors initially identified by Price’s Law (Figure 4). This chronological visualization maps the evolution of these research communities, revealing three distinct generational shifts from early physical exploration to modern clinical and engineering applications.

**Figure 4:**
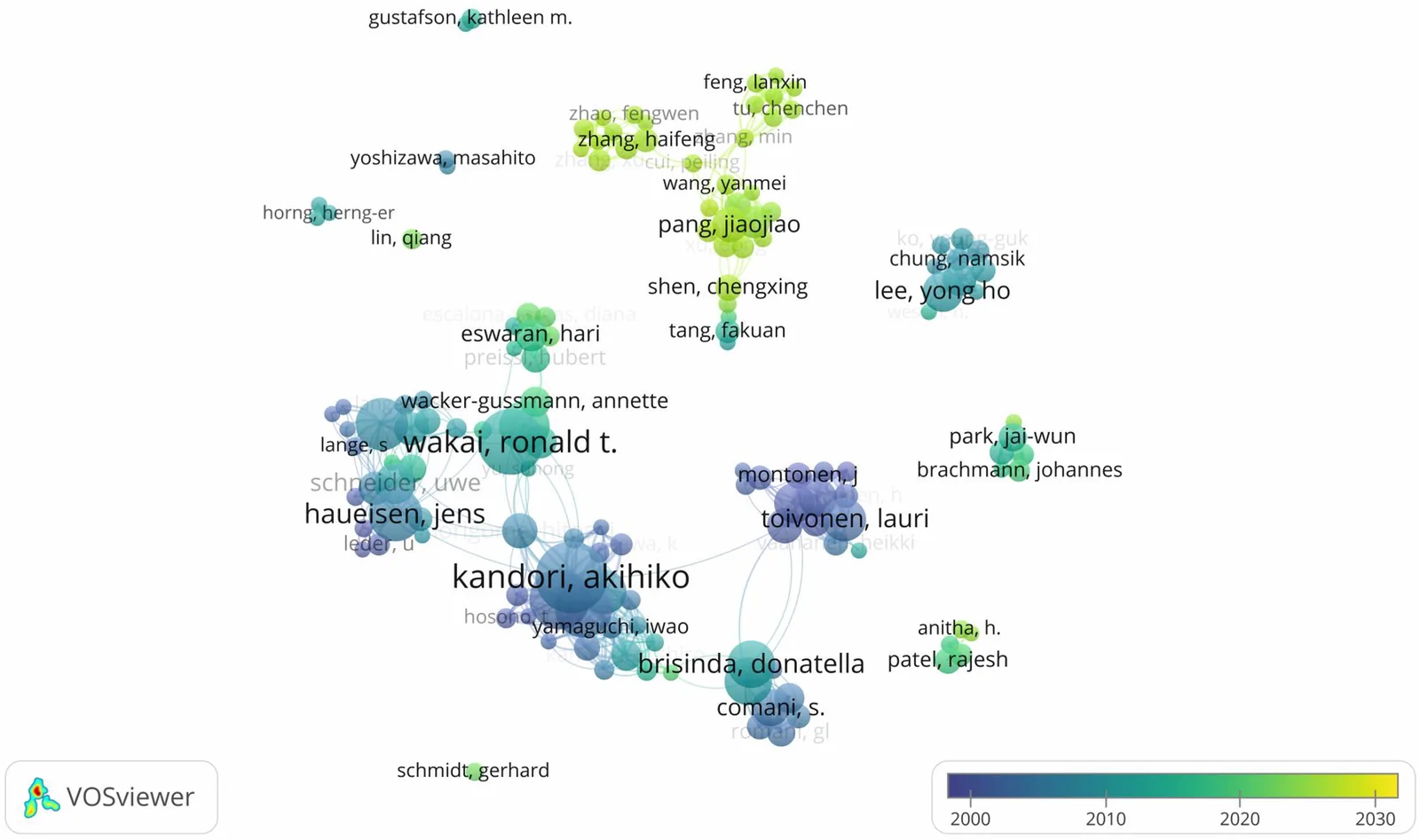
Time-overlay co-occurrence network of core authors (Generated using VOSviewer).

The foundational phase, represented by the dark blue nodes, was spearheaded by European pioneers such as T. Katila from Aalto University. Active since the 1970s, this group established the theoretical and physical groundwork for acquiring biomagnetic signals using SQUID. Their seminal contributions include early fetal MCG recordings (17, 18), the modeling of current dipoles in realistic torsos (19), and the experimental validation of vortex currents causing MCG and ECG signal divergences (20), cementing the physical viability of MCG.

Subsequently, the translational phase (teal nodes) highlights the shift from basic physical research to clinical applications. This period was largely led by A. Kandori from Hitachi and R. T. Wakai from the University of Wisconsin-Madison. A. Kandori, the most prolific author in the field (41 documents), extensively explored the clinical utility of MCG across fetal monitoring (21, 22), CAD (23), and arrhythmias (24). Concurrently, R. T. Wakai of the University of Wisconsin-Madison and J. F. Strasburger of the Medical College of Wisconsin played pivotal roles in fetal MCG (21). J. F. Strasburger holds the highest total citations (1,751) within the network. While both researchers significantly advanced the application of OPM arrays for fetal assessment (25), J. F. Strasburger notably contributed to the establishment of authoritative clinical statement, including the American Heart Association scientific statement on fetal cardiac disease (26).

As illustrated by the yellow-green nodes, this cluster represents an active contemporary research frontier. This phase is characterized by a community of Chinese scholars, with researchers such as X. Ning from Beihang University, and J. Pang from Qilu Hospital of Shandong University making notable contributions. As reflected in recent literature, this group’s high productivity is closely associated with advancements in ultra-weak magnetic sensing, specifically the deployment of spin-exchange relaxation-free (SERF) OPM systems. Their research encompasses hardware system optimization (27), advanced signal preprocessing and extraction (28, 29), magnetic source localization (30, 31), and subsequent clinical applications, including non-invasive assessments of CAD (32, 33). These contributions are facilitating the continuous technological optimization of MCG and its practical clinical implementation.

### 3.4 Journals and co-occurrence network

Applying Bradford’s Law, the 183 source journals were stratified to identify the most concentrated publication venues. The analysis identified a core zone of 10 journals that collectively published 186 articles, accounting for 29.48% of the total literature. This distribution is consistent with the theoretical expectation of Bradford’s Law, which posits that the core zone should contain approximately one-third of the total publications (34). As detailed in Table 4, the top five most prolific journals are *Physiological Measurement* (27 publications), *Pacing and Clinical Electrophysiology* (24 publications), and a three-way tie among *Annals of Noninvasive Electrocardiology*, *IEEE Transactions on Biomedical Engineering*, and *Physics in Medicine and Biology* (each with 20 publications). These key journals reflect that MCG research spans multiple disciplines, including clinical cardiology, physics, and biomedical engineering.

**Table 4:** Ranking of the top 10 core journals (Source: VOSviewer).

Rank	Source	Publications	Citations	Average citation/publication
1	Physiological Measurement	27	463	17.15
2	PACE-Pacing and Clinical Electrophysiology	24	436	18.17
3	Annals of Noninvasive Electrocardiology	20	304	15.20
4	IEEE Transactions on Biomedical Engineering	20	440	22.00
5	Physics in Medicine and Biology	20	542	27.10
6	IEEE Transactions on Instrumentation and Measurement	18	336	18.67
7	Sensors	16	248	15.50
8	Journal of Electrocardiology	15	186	12.40
9	Biomedical Signal Processing and Control	13	107	8.23
10	IEEE Transactions on Applied Superconductivity	13	147	11.31

To further map the intellectual structure of the publication sources, a journal co-occurrence network was constructed (Figure 5). Based on thematic similarities and citation linkages, the journals are classified into three distinct disciplinary clusters, reflecting the diverse application trajectories of MCG technology.

**Figure 5:**
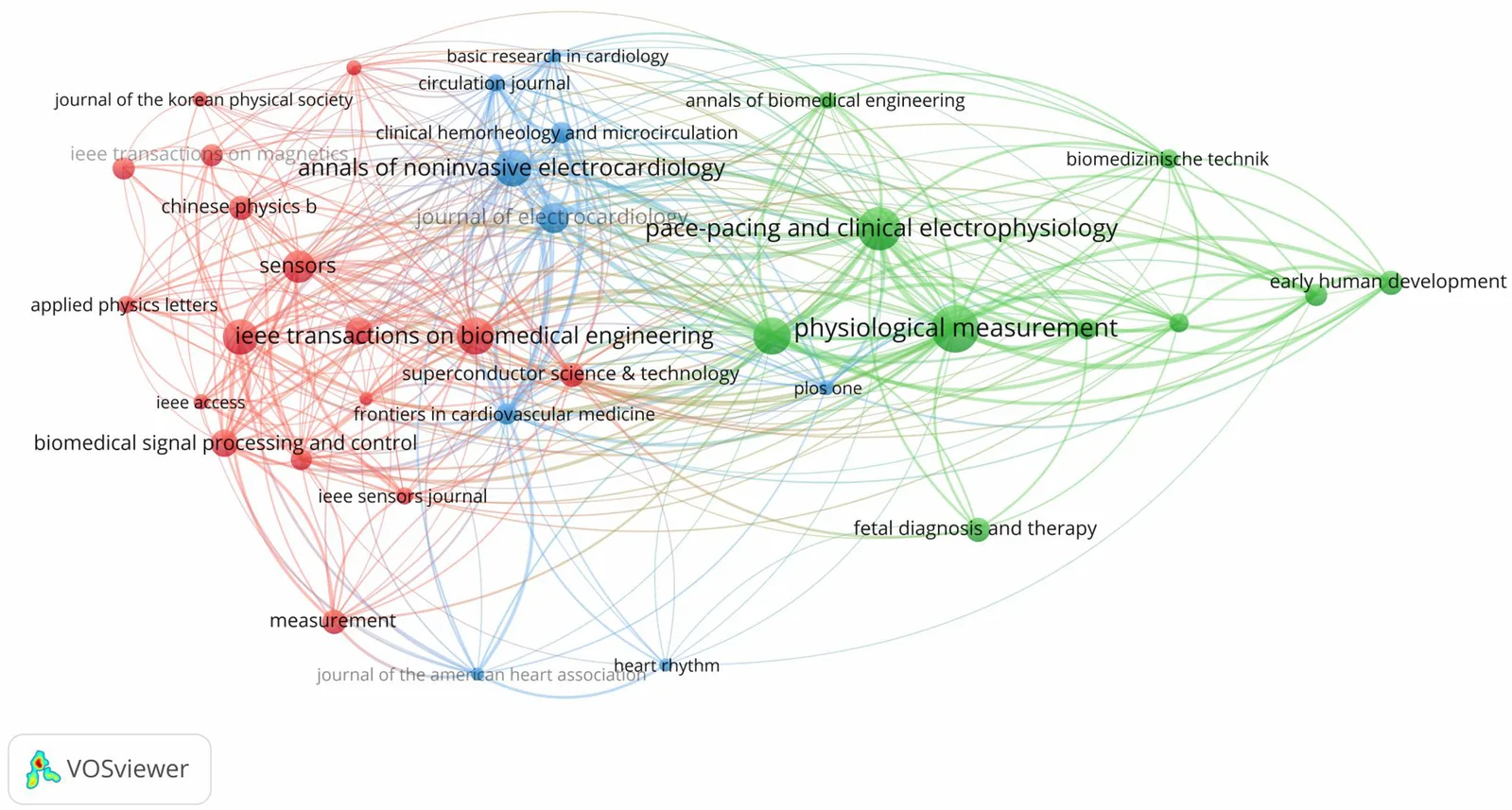
Journal co-occurrence network (Generated using VOSviewer).

The red cluster forms the engineering and sensor technology foundation, primarily focusing on hardware development and fundamental signal processing. This cluster is anchored by prominent journals such as *Sensors*, *IEEE Transactions on Biomedical Engineering*, and *IEEE Transactions on Instrumentation and Measurement*. Highly cited papers within this group predominantly consist of focused literature reviews on specific sub-domains or studies presenting methodological innovations (35–38). Concurrently, recent publications in these journals highlight current research priorities, including the optimization of sensor array geometries (25, 39), the design of self-shielding coils (40), and the diversification toward integrated SERF OPM systems (41).

The green cluster represents the intersection of physics and physiological measurement, with a particularly strong focus on maternal-fetal medicine and pediatric applications. Core journals in this domain include *Physiological Measurement*, *Pacing and Clinical Electrophysiology (PACE)*, and *Physics in Medicine and Biology*. The historical impact of this cluster is deeply rooted in fetal electrophysiology, featuring highly cited guidelines for fetal MCG standards and early development in extracting fetal cardiac signals via independent component analysis (ICA) (42–44). More recently, research in these journals has shifted toward evaluating compact OPM arrays to detect heart disease in animals (45) and track fetal maturation and diagnose congenital arrhythmias, such as long QT syndrome (46).

The blue cluster signifies the clinical translation of MCG into mainstream cardiology, focusing on its utility as a non-invasive diagnostic tool. Centered around clinical journals like the *Annals of Noninvasive Electrocardiology* and the *Journal of Electrocardiology*, this cluster is dominated by clinical trials and observational cohort studies. The highly cited papers primarily focus on elucidating MCG technology and its clinical applications in myocardial ischemic diseases (47–50). Recent literature within this cluster continues to expand MCG’s clinical footprint, exploring its prognostic value in evaluating sudden cardiac death risk (51), monitoring recurrent angina following percutaneous coronary intervention (52), and assessing neonatal autonomic functions (53).

### 3.5 Knowledge mapping and topic evolution

To systematically understand the conceptual structure and historical trajectory of MCG research, keyword co-occurrence and timeline analyses were conducted. While static keyword clustering maps can delineate thematic boundaries, they often lack temporal depth and can create visual redundancy when paired with timezone analyses. Therefore, to optimize the analytical framework, this study utilized a VOSviewer co-occurrence network (Figure 6) alongside CiteSpace timeline (Figure 7) and citation burst analyses (Figure 8). This combination effectively maps both the spatial distribution of current knowledge and the dynamic temporal evolution of research frontiers. Although the static clustering map was omitted, the thematic groupings presented in the CiteSpace timeline visualization (Figure 7) are generated by an underlying clustering algorithm. The mathematical validity of this clustering is confirmed by a Modularity Q score of 0.8069 (where Q>0.3 indicates significant community structure) and a Weighted Mean Silhouette S score of 0.9291 (where S>0.7 indicates reliable clustering).

**Figure 6:**
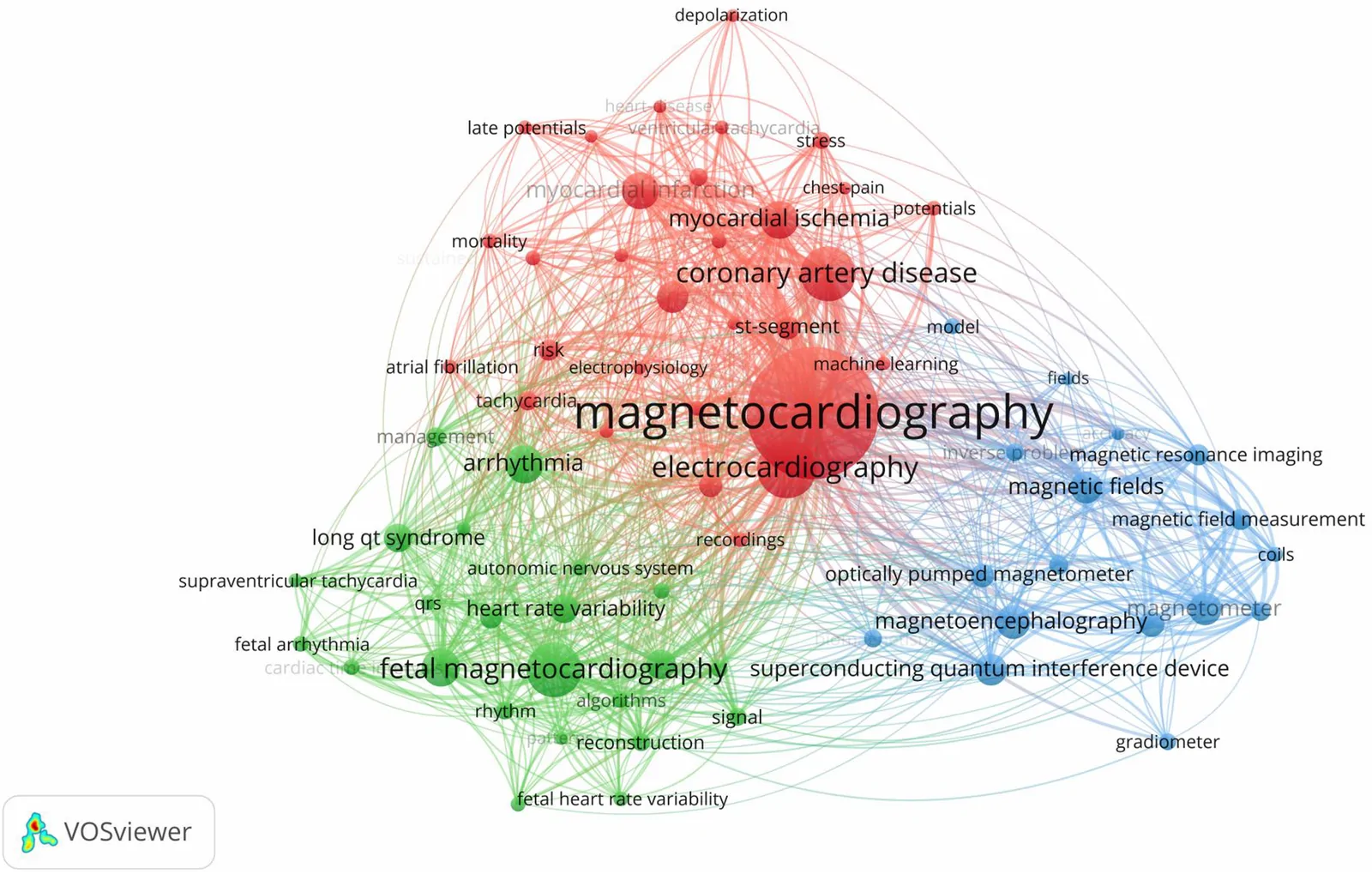
Keyword co-occurrence network mapping the core research topics (Generated using VOSviewer).

**Figure 7:**
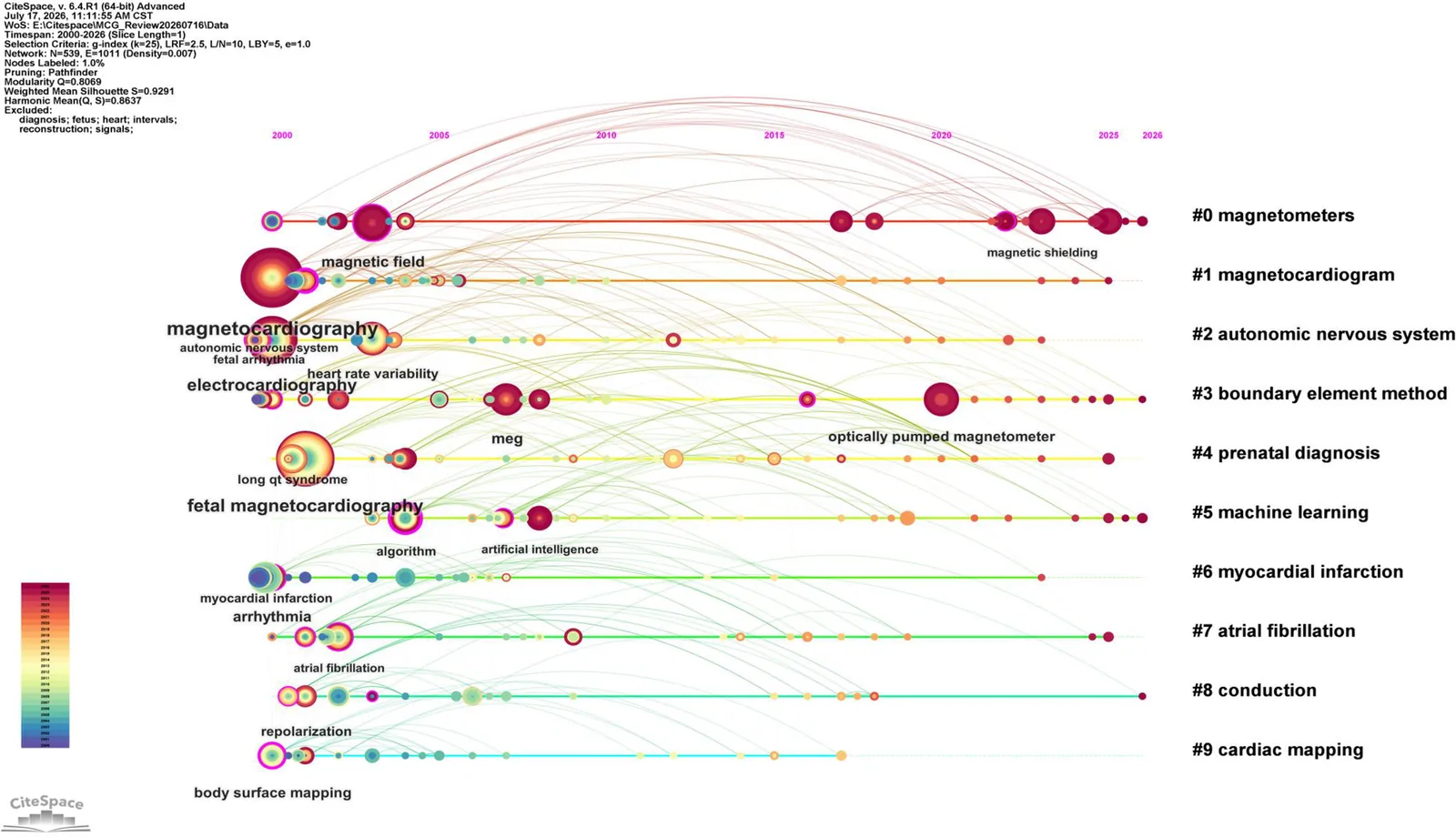
Keyword timeline view illustrating the evolutionary trajectory of research hotspots over time (Generated using CiteSpace).

**Figure 8:**
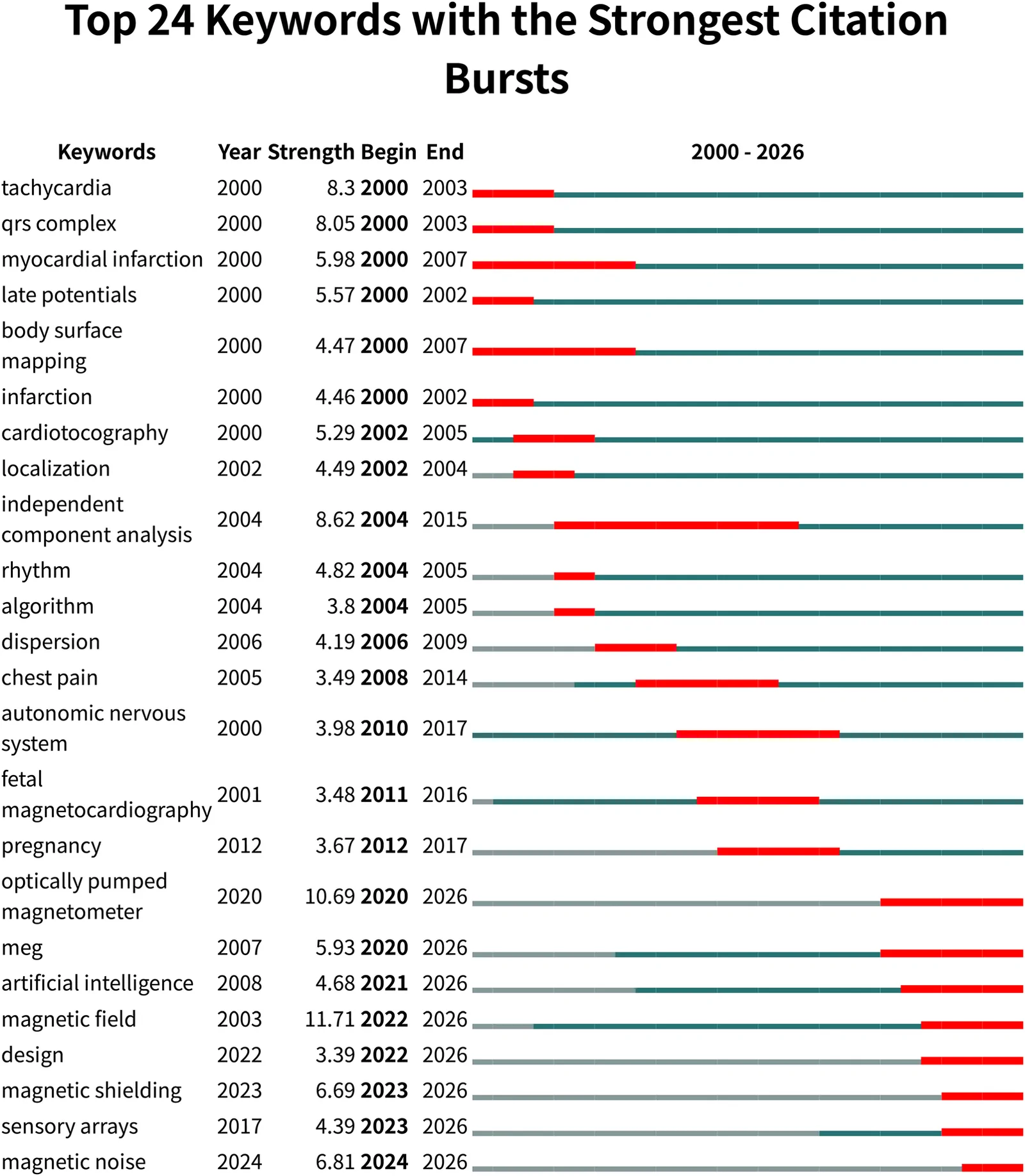
Keyword burst detection map indicating the intensity and duration of emerging research trends (Generated using CiteSpace).

The VOSviewer co-occurrence network (Figure 6) reveals three interwoven yet distinct primary research domains. Notably, the core term “MCG” occupies the structural center of the network, acting as a pivotal hub that interconnects these three disciplines. The hardware and physics domain (blue cluster) is centered around terms such as “magnetometers,” “SQUID,” “OPM,” and “magnetic fields.” This cluster focuses on the foundational instrumentation required to capture ultra-weak biomagnetic signals and extract fundamental physiological parameters within high magnetic noise environments. The adult clinical diagnosis domain (red cluster), anchored by “MCG,” “myocardial ischemia,” and “CAD”, represents the clinical translation pathway, utilizing MCG to non-invasively detect ischemic heart disease. Lastly, the fetal monitoring and electrophysiology domain (green cluster) is dominated by “fetal MCG,” “heart rate variability,” and “autonomic nervous system (ANS).” Heart rate variability has emerged as a vital tool reflecting ANS regulation (54).

To systematically map the chronological evolution of these domains and capture sudden surges in research interest, a CiteSpace burst detection analysis (Figure 8) was integrated with the timeline view (Figure 7) (11, 13). This combination helps identify temporal shifts in research focus and highlights emerging hotspots across different developmental stages.

The first phase (2000–2007) reflects a period of preliminary clinical exploration. During this period, the strongest citation bursts included keywords such as “myocardial infarction” (strength 5.98), “qrs complex” (8.05), “body surface mapping” (4.47), and “tachycardia” (8.30). The literature in this stage primarily focused on phenomenological validation. Specifically, early studies mapped magnetic field distributions to establish diagnostic comparisons with traditional ECG features, particularly concerning infarction and tachycardia (55, 56).

The second phase (2010–2017) transitioned into a stage characterized by the integration of signal separation techniques with specialized clinical applications. Bursts during this mid-term period were dominated by terms like “ICA” (8.62), “fetal MCG” (3.48), and “ANS” (3.98). The concurrent emergence of these keywords highlights a growing intersection between computational algorithms and clinical assessments within the literature. Accordingly, studies during this timeframe frequently utilized blind source separation methods to isolate ultra-weak cardiac signals, supporting high-resolution assessments of fetal electrophysiology and autonomic nervous system parameters (21, 57–59).

The third and current phase (2020–2026) represents a contemporary technological development centered on novel sensing and computational methods. This phase is characterized by bursts in keywords such as “OPM” (10.69), “artificial intelligence (AI)” (4.68), “magnetic shielding” (6.69), “sensory arrays” (4.39), and “magnetic noise” (6.81). Reflected by these prominent terms, the primary research frontiers in this ongoing stage encompass the development of room-temperature sensory array layouts and the advancement of magnetic shielding technologies (25, 60, 61). Beyond these hardware topics, recent articles focus on refining AI algorithms to improve data preprocessing and the accuracy of clinical classification (32, 62, 63).

### 3.6 Co-citation analysis

Co-citation analysis maps the intellectual foundations and interdisciplinary knowledge flows that underpin a research field. To deconstruct the conceptual base of MCG, a reference co-citation network (Figure 9) was initially constructed, revealing three distinct but synergistic thematic clusters. The green cluster forms the foundational and classical clinical base, anchoring the field’s origins from pioneering physical detections using early SQUID in shielded environments (8, 64) to early clinical validations for adult ischemic heart disease. Seminal works here firmly established the diagnostic value of MCG in detecting acute chest pain and CAD relative to traditional ECG (49, 65–67). Conversely, the red cluster delineates a specialized and impactful clinical sub-domain: fetal MCG. Because traditional fetal ECG is heavily distorted by maternal tissues, MCG provides an unique advantage for prenatal electrophysiology. Core literature in this cluster focuses on defining fetal heart rate variability, arrhythmias, and developmental cardiac intervals (65, 68–70). The blue cluster represents a contemporary technological frontier. Recent literature in this cluster increasingly addresses room-temperature OPM instrumentation (71), while SQUID-based systems remain an important component of the field (38).

**Figure 9:**
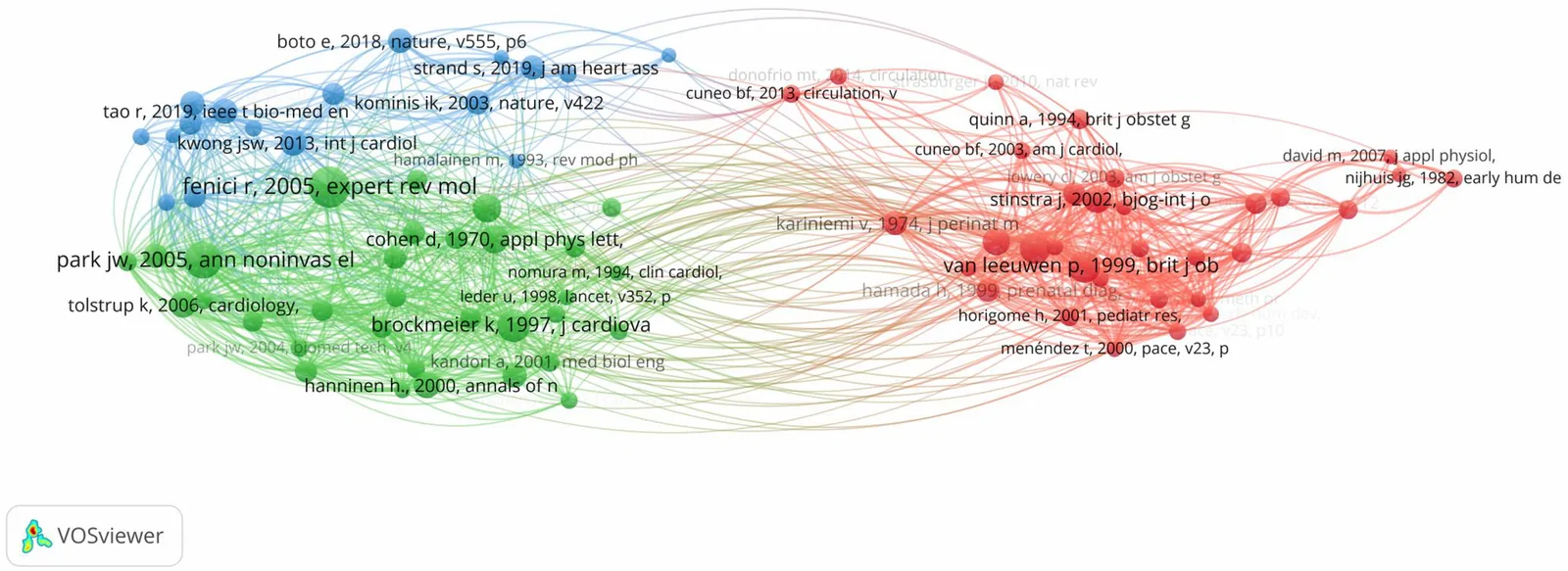
The co-citation network of references detailing foundational knowledge clusters (Generated using VOSviewer).

The intellectual structure of these domains is further elucidated by the source (journal) co-citation network (Figure 10). Spatially, this network illustrates a structural progression from fundamental engineering research to clinical applications. The left-sided red cluster (physics and engineering technology) provides the sensory and metrological hardware foundation. The right-sided green cluster (clinical and maternal-fetal medicine) represents the diagnostic application endpoints. The centrally located blue cluster (cardiac electrophysiology) functions as an intermediate bridge. It connects engineering advancements with physiological applications, facilitating the transfer of physical measurement techniques into clinical practice.

**Figure 10:**
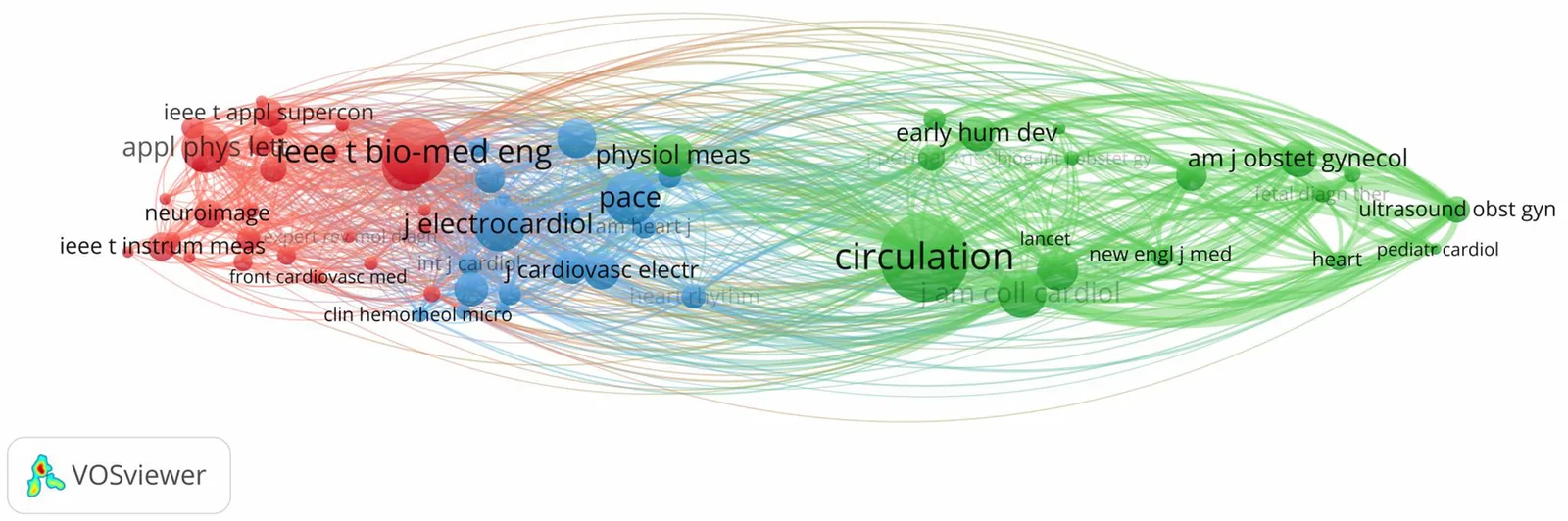
The co-citation network of sources representing the structural relationship among key journals (Generated using VOSviewer).

This interdisciplinary integration is inherently reflected in the author co-citation network (Figure 11), which exhibits a pronounced center-periphery topology with no isolated nodes. The network is organized by multiple core hubs, notably R. Fenici (blue cluster), whose central positioning and seminal reviews on clinical MCG utility, serve as the primary structural bridge for the entire network (4, 48, 67). To the right, the red cluster is heavily anchored by P. Van Leeuwen, whose highly cited works define the standards for analyzing fetal autonomic functions and maternal-fetal coordination (72–74). The green cluster features foundational pioneers like D. Cohen, who established the theoretical basis for mapping torso magnetic fields (75, 76), alongside modern clinical leaders like J. W. Park, who cemented MCG’s predictive value in adult CAD (49, 77). The high density of inter-cluster linkages indicates active interdisciplinary knowledge exchange across the field, from physical instrumentation to clinical practice.

**Figure 11:**
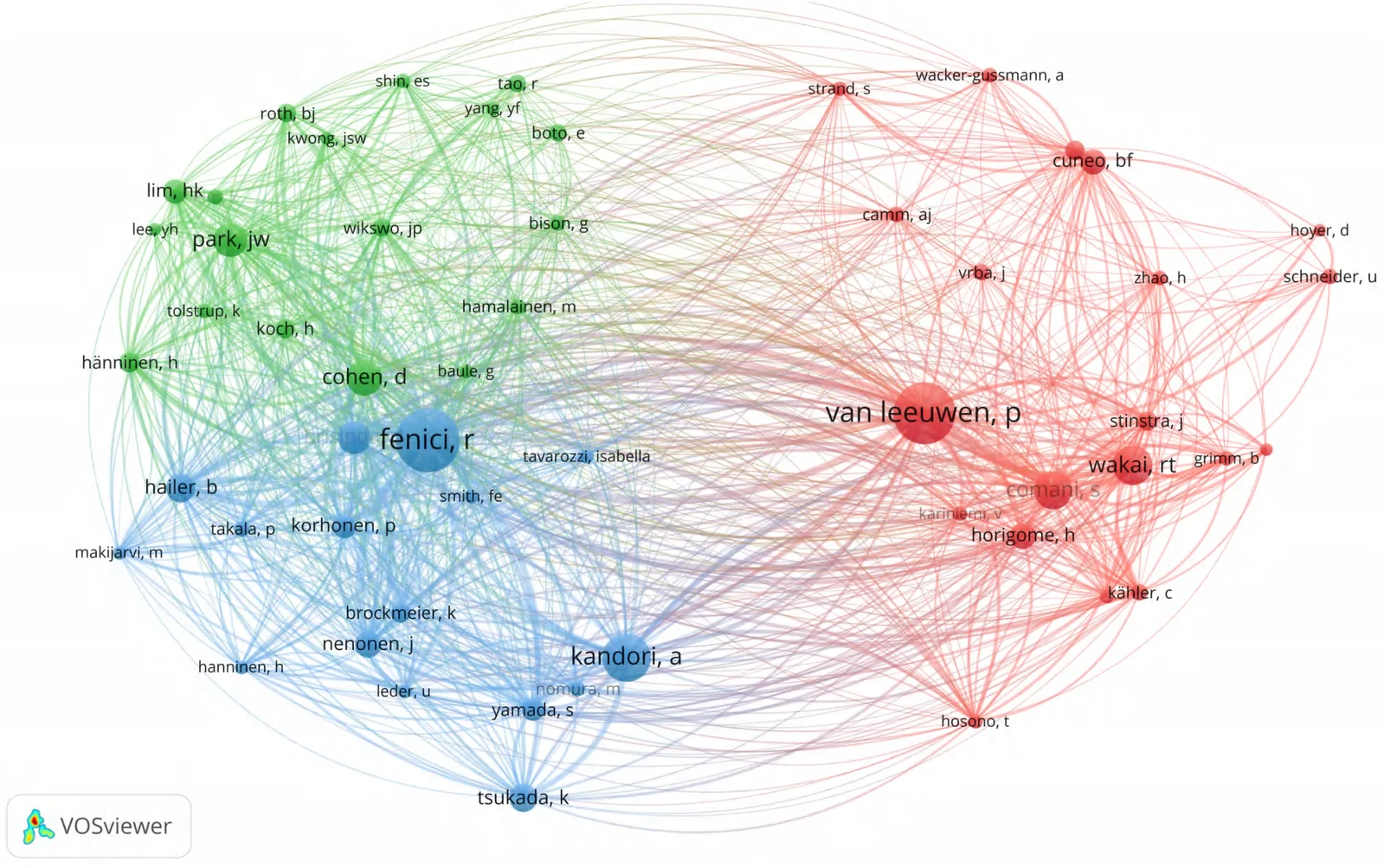
The co-citation network of authors revealing the most influential contributors in the field (Generated using VOSviewer).

Finally, the dual-map overlay of journals (Figure 12) visualizes the knowledge flow and interdisciplinary nature of the field. In this map, the right side represents the cited journals (the intellectual foundation), while the left side denotes the citing journals (the current research front) (78). The visualization reveals a clear converging pattern, primarily highlighted by the thick green connecting lines. Specifically, foundational research from disciplines on the right, such as Health/Nursing/Medicine and Molecular/Biology/Genetics, flows directly into a concentrated clinical application hub on the left (Medicine/Medical/Clinical). Furthermore, supplementary citation paths originating from Physics/Materials/Chemistry on the right demonstrate the important hardware and engineering support for this field. Overall, this converging structure provides clear evidence that modern MCG is an interdisciplinary field, relying heavily on the combination of physical engineering, biological mechanisms, and medical science to advance clinical diagnostics.

**Figure 12:**
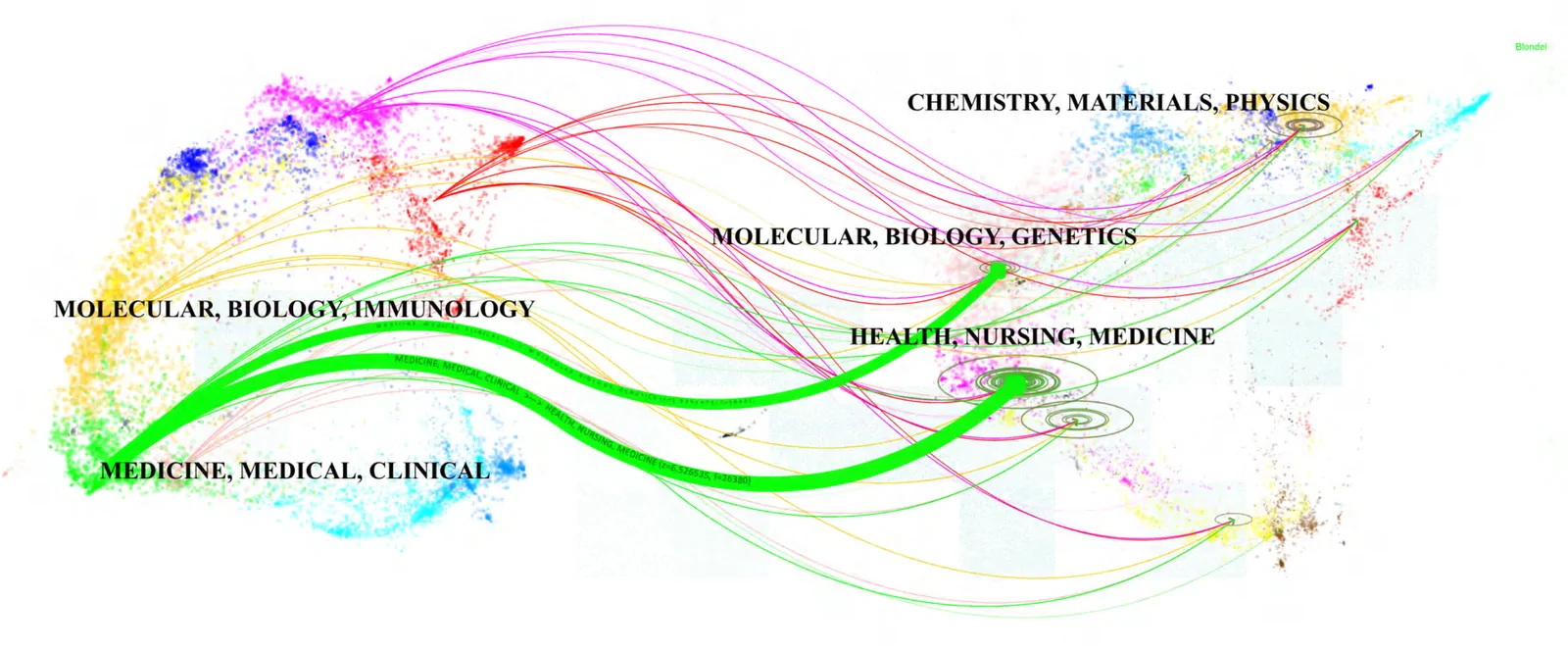
Dual-map overlay of co-cited journals highlighting citation trajectories across different disciplinary domains (Generated using CiteSpace).

### 3.7 Scopus database validation

To ensure the methodological robustness and reliability of the bibliometric findings derived from the WoSCC, a comparative sensitivity analysis was conducted using the Scopus database. As established in the inclusion criteria, an initial cross-database assessment confirmed a substantial literature overlap, with 548 shared documents representing a 65.4% overlap of the merged dataset (*n* = 838). This substantial overlap provides an independent robustness check for the primary WoSCC-based analysis and suggests that the major temporal patterns are largely insensitive to database selection.

The temporal consistency of the field’s evolution is corroborated by comparing the annual publication trends across both repositories (Figure 13). The publication trajectories from WoSCC (blue curve) and Scopus (orange curve) exhibit synchrony over the past 26 years. Both curves accurately capture the prolonged mid-term plateau, the noticeable nadir around 2021, and the subsequent substantial growth trajectory that culminated in a historical peak in 2025. The temporal consistency verifies that the multiphasic evolution of MCG research is a robust, database-independent phenomenon. Notably, the recent publication surge, coinciding with parallel advancements in OPM instrumentation and AI-based signal analysis, suggests an expansion of research activity rather than a database-specific indexing artifact.

**Figure 13:**
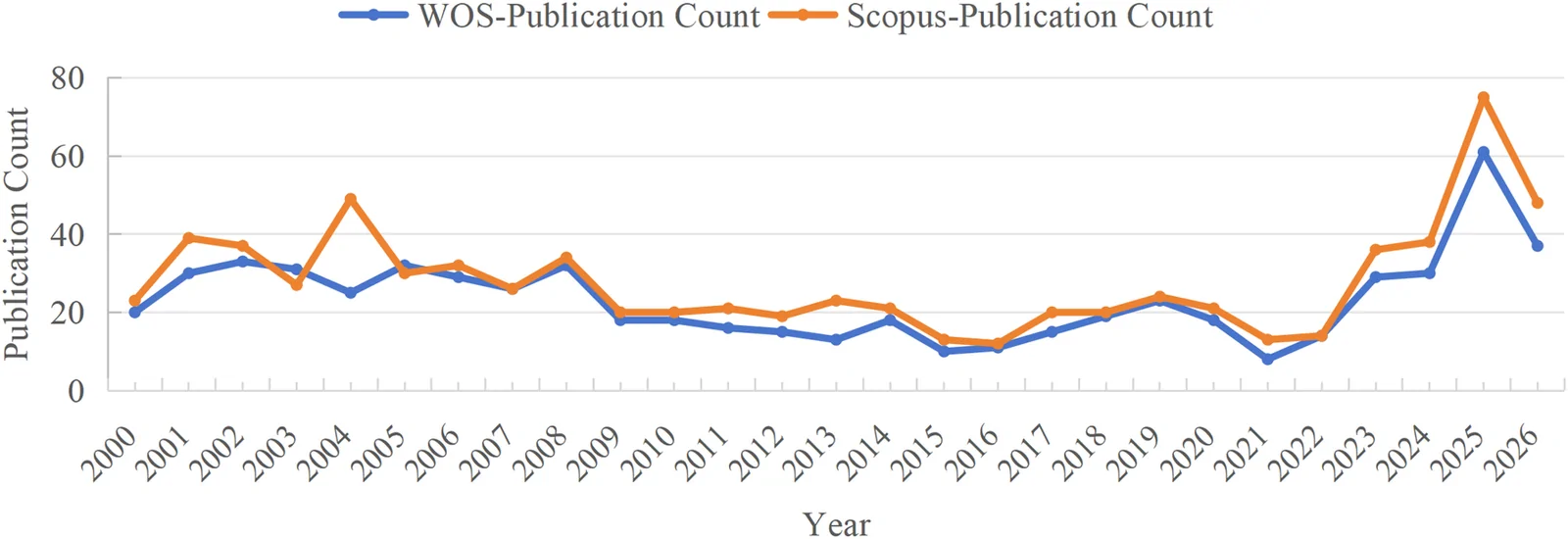
Comparison of annual publication trends between the WoSCC and Scopus databases.

A comparative analysis of source coverage was performed to evaluate potential disciplinary distribution biases (Figure 14). The journal overlap analysis (Figure 14A) illustrates that 173 journals (67%) are mutually indexed, effectively capturing the conceptual core of the field. While Scopus encompasses a larger proportion of uniquely indexed journals (30%) compared to WoSCC (4%), the side-by-side distribution of the core journals (Figure 14B) reveals a strict thematic alignment. Both databases consistently identify *Physiological Measurement*, *Pacing and Clinical Electrophysiology*, and *IEEE Transactions on Biomedical Engineering* as the primary publication venues. Notably, Scopus exhibits higher document counts in certain specialized clinical journals, such as *Neurology and Clinical Neurophysiology* and *Am. Heart J. Plus*. These variations reflect Scopus’s inherently broader coverage of specific medical sub-disciplines rather than a fundamental divergence in the field’s core focus. The core engineering and cardiovascular clinical venues remain structurally consistent across both platforms. Consequently, these validation findings indicate that the primary bibliometric conclusions, knowledge networks, and evolutionary trends derived from the WoSCC dataset are scientifically robust and representative.

**Figure 14:**
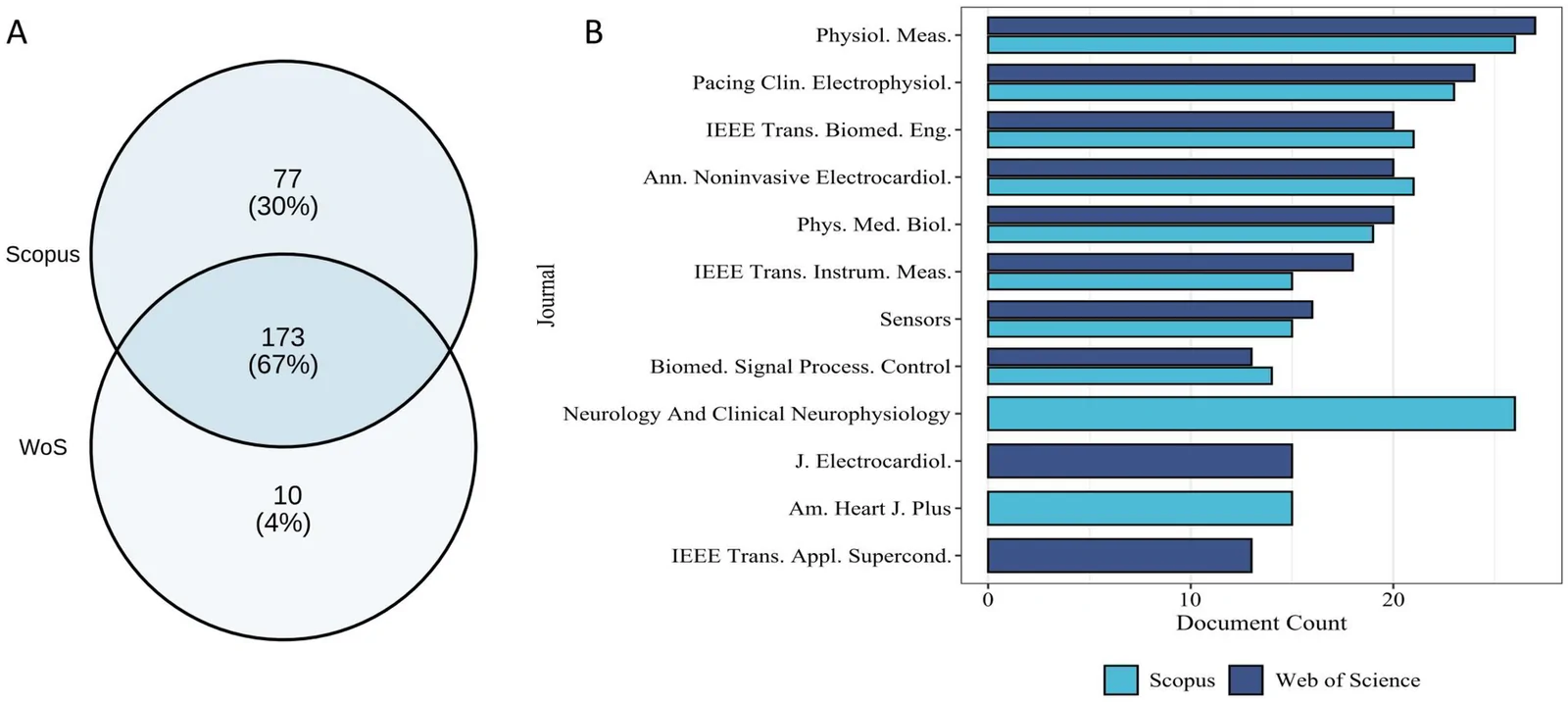
Comparison of journal coverage and document distribution between WoSCC and Scopus. **(A)** Venn diagram illustrating the intersection and database-specific journal counts. **(B)** Document counts of the top high-yield journals, stratified by database indexing.

## 4 Discussion

The findings reveal that MCG is an expanding, interdisciplinary field, characterized by the parallel development of hardware innovation, AI-based analysis, and clinical applications.

### 4.1 General information and global trends

Since 2000, the annual publication trajectory of MCG research has been multiphasic, reflecting an ongoing interplay between technological bottlenecks, translational barriers, and subsequent breakthroughs. The prolonged stagnation and noticeable nadir around 2021 may reflect several interacting translational barriers. These may have included the infrastructure demands of legacy cryogenic systems, a lack of standardized multicenter validation, reimbursement challenges, and difficulties in clinical workflow integration. Such hurdles were compounded by the COVID-19 pandemic, which disrupted clinical trials and hardware supply chains (79, 80). The subsequent surge, peaking in 2025, reflects concurrent advances in OPM-based instrumentation, AI-based signal analysis, and magnetic-field control. Clinical translation has also progressed, notably the regulatory clearance of commercial MCG systems (81).

Geographically, Germany produces the most publications in this field. It is noteworthy that high publication volume does not strictly equate to high citation impact, as seen in the comparison between China (high output) and the Netherlands (high average citations). This divergence may suggest that while some countries account for a substantial publication volume, others contribute influential or seminal papers to the field. The leading positions of Western countries are likely related to early national investments in expensive magnetic shielding facilities necessary for foundational biomagnetic research (82, 83). These geographic patterns may reflect differences in research funding, domestic laboratory infrastructure, and international collaboration networks. Interestingly, as evidenced by the deviation from Price’s Law identified in the author distribution, the core researcher community remains unconsolidated. This multidisciplinary nature may inherently foster a diversified, broad-ranging collaborative network. For example, physicists design the quantum sensors, algorithm engineers process the signals, and cardiologists evaluate the clinical outcomes (67, 83).

The conceptual structure of MCG research is grounded in three interdependent knowledge domains that collectively contribute to its translational advancements. First, the “hardware and physics” domain forms the foundational layer, focusing on extracting ultra-weak biomagnetic signals from noisy environments through innovations in sensor technology. This domain is currently characterized by the parallel optimization of mature, high-fidelity SQUID systems and the development of emerging, cryogen-free OPM technologies. Building on this physical foundation, the “adult clinical diagnosis” domain applies MCG for the non-invasive assessment of ischemic conditions. Concurrently, the “fetal monitoring and electrophysiology” domain leverages MCG’s distinct immunity to maternal tissue interference, enabling monitoring of fetal cardiac rhythms. Notably, these fundamental advancements in hardware and physics lay the groundwork for clinical applications in the other two domains. This creates an interdependent research structure where technological innovations and diagnostic applications reinforce each other, establishing a continuous translational pathway for MCG.

### 4.2 Research hotspots and thematic evolution

Keyword-burst analysis identifies a three-phase research timeline. Underlying research priorities evolve from phenomenological validation toward advanced signal extraction. Most recently, technology-oriented studies have sought to address practical and analytical obstacles hindering the clinical translation of MCG.

The first phase (2000–2007) was characterized by a fundamental need to establish clinical credibility. Keywords such as “QRS complex” reflect the field’s initial reliance on morphological comparisons between MCG and the gold-standard ECG. During this period, studies conducted largely with established SQUID-based systems focused on mapping established cardiac electrical events, such as QRS morphology, onto magnetic-field distributions (69, 84). This phase was primarily focused on exploring the diagnostic characteristics of MCG and comparing its performance relative to established clinical tools like ECG.

As the fundamental viability of MCG was confirmed, the second phase (2010–2017) shifted toward overcoming the inherent signal-to-noise ratio limitations of biomagnetic measurements. The burst of “ICA” marks a critical algorithmic inflection point. Researchers increasingly adopted blind source separation frameworks, particularly ICA, to recover original sources from mixed signals (85). By mathematically isolating independent components, this method provided an approach to separating cardiac sources from background interference (85, 86). This algorithmic advancement coincided with the improved extraction of ultra-weak fetal cardiac signals, which are otherwise masked by the dominant maternal heartbeat and volume conduction artifacts (87–89). Thus, this phase marked a shift from basic signal detection toward improved signal fidelity.

The third and current phase (2020–2026) represents a technology-driven expansion of MCG, with increasing attention to the practical acquisition of high-dimensional cardiac magnetic signals and environmental-field control. Although the conceptual application of OPM for MCG was demonstrated as early as 2003 (90), the keyword “OPM” did not exhibit a citation burst until 2020. This temporal separation suggests a prolonged period of engineering development between early proof-of-principle demonstrations and broader research adoption. Recent studies increasingly focus on SERF and other room-temperature OPM implementations, including multichannel array design and system integration (91–93). However, the practical implementation of OPM-based MCG remains subject to architecture- and system-dependent constraints, including dynamic range, residual-field sensitivity, field zeroing and calibration requirements, cross-axis errors, sensor crosstalk, and motion-related signal perturbations (83, 91). The concurrent emergence of “magnetic shielding”, “sensory arrays”, and “magnetic noise” further indicates that recent technological efforts are directed not simply toward changing the sensor platform, but toward improving field control, spatial coverage, signal robustness, and ultimately the practicality of MCG acquisition (29, 94). Emerging alternatives such as TMR and NV-center magnetometers may further diversify the future sensor landscape (95, 96).

From an economic perspective, a direct cost comparison between SQUID and OPM systems is complex and requires evaluating the overall system expenses. OPMs offer the clear advantage of cryogen-free operation, but this alone does not guarantee a lower overall system cost. Multichannel OPM systems still entail substantial upfront sensor costs and demanding supporting infrastructure, including high-performance magnetic shielding, active field compensation, and routine calibration (97). Traditional SQUID systems, by contrast, incur high liquid-helium costs, but modern installations can substantially reduce these recurring expenses through integrated cryocoolers and continuous helium recovery systems (98). Therefore, generalized claims regarding the absolute cost-effectiveness of either technology should be interpreted with caution, as the underlying economics heavily depend on specific sensor configurations, infrastructure readiness, and target clinical applications.

Parallel to these hardware innovations, the increasing burst of “AI” highlights a crucial, yet partly independent, analytical trajectory. Technological development has extended beyond sensor hardware to the automated extraction of high-dimensional clinical information, with recent studies applying machine learning and deep learning methods for feature extraction, classification, and clinical prediction (38, 99). Importantly, this analytical evolution is not exclusively a consequence of the transition to OPMs; high-performance AI-MCG studies continue to be successfully executed using mature SQUID-based systems (63). This reflects a contemporary research landscape where hardware innovation and sophisticated data analysis evolve concurrently to overcome the historical barriers of clinical MCG implementation.

### 4.3 Clinical applications and future challenges

As mapped by the VOSviewer co-occurrence network (Figure 6), the clinical translation of MCG has predominantly focused on specific cardiovascular domains. The adult clinical diagnosis domain highlights applications in ischemic heart disease and arrhythmias, while a distinct domain is dedicated to fetal electrophysiology. Importantly, the clinical evidence underlying these application domains is technologically heterogeneous. Representative clinical and translational studies, stratified by sensor technology, magnetic environment, analytical approach, and evidence level, are summarized in Table 5.

**Table 5:** Technology-stratified summary of representative clinical and translational magnetocardiography studies.

Study	Clinical application	Population/N	Sensor tech.	Sensor configuration	Magnetic environment	Analysis	Reference standard	Principal result	Evidence level	SQUID–OPM comp.
Kanzaki et al., 2003 (23)	CAD screening	30 subjects (17 CAD)	SQUID	64-channel MCG (MC-6400)	MSR	QRS integral change	CAG	82% sens., 85% spec. for CAD detection.	Prospective clinical	No
Park et al., 2005 (49)	CAD in acute chest pain	185 analyzed patients	SQUID	9-channel SQUID mapped to 36 positions	Unshielded	Visual assessment, automated parameters	CAG	Higher sens. and NPV than admission ECG, ECHO, and troponin.	Prospective clinical	No
Steinberg et al., 2005 (50)	Ischemia detection	53 subjects (29 suspected CAD)	SQUID	9-channel SQUID + 3 ref. channels	Unshielded	Contour maps, waveform morphology	CAG	Reproducible Unshielded acquisition; 84.2% sens. for ischemia (vs. 26.3% ECG).	Clinical feasibility	No
Lim et al., 2009 (66)	NSTE-ACS detection	397 subjects (110 UAP, 83 NSTEMI)	SQUID	64-channel MCG	MSR	Conventional MCG parameters	CAG & clinical assessment	Abnormal maps in 70% UAP, 92.5% NSTEMI; combined parameters 96.4% sens.	Clinical cohort	No
Cuneo et al., 2013 (21)	Fetal LQTS/arrhythmia	30 fetuses at risk for LQTS	SQUID	37- and 64-channel MCG systems	MSR	Waveform morphology, QTc, rhythm	Neonatal ECG, genetics	QTc ≥ 490 ms: 89% sens./spec. for LQTS; QTc ≥ 620 ms associated with TdP.	Retrospective clinical	No
Alem et al., 2015 (59)	Fetal MCG feasibility	1 pregnant woman	OPM	25 microfabricated OPMs in conformal array	MSR	Orthogonal projection, ICA, FHR extraction	NR	Successfully separated maternal/fetal signals and extracted FHR.	Proof-of-concept	No
Strand et al., 2019 (71)	Fetal rhythm	24 pregnant subjects	Both	SQUID: 7-channel vector (21 sensors); OPM: 10 sensors	SQUID-MSR; OPM-CS & MSR	Spatial filtering, SNR comparison	Fetal ECHO & clinical assessment	No significant SNR difference; both systems produced recordings considered adequate for fetal rhythm assessment.	Comparative feasibility	Yes
Tao et al., 2019 (38)	IHD detection/localization	574 subjects (227 coronary stenosis)	SQUID${}^{a}$	4-channel MCG system	Unshielded	ML (KNN, DT, SVM, XGBoost)	CAG	IHD detection: 94% acc., AUC 0.98. Localization acc.: 0.74 (LAD), 0.68 (LCX).	Retrospective clinical	No
Han et al., 2023 (32)	CAD severity/localization	309 patients with chest pain	OPM	36-channel MCG system	Semi-open shield	ML (1D features)	CAG or CTA	SVM AUC 0.876 for CAD severity; localization ≥ 84.4% for LAD/LCX/RCA.	Prospective clinical	No
Han et al., 2023 (45)	Acute MI/PVC detection	4 dogs	OPM	36-channel MCG system	Multilayer CS	Magnetic field maps, PCD maps	LAD ligation, ECG, cardiac enzymes	Demonstrated detection of acute MI and PVC-related magnetic changes.	Animal model	No
Kranz et al., 2026 (63)	CAD, LVEF, AF risk	1,732 consecutive patients	SQUID	64-channel SQUID array	MSR	Deep learning (contrastive learning)	CAG, ECHO, clinical history	AUC 0.89 for multivessel CAD, 0.81 for reduced LVEF, 0.77 for AF risk.	Retrospective clinical + AI	No
Lachlan et al., 2026 (101)	SCD/ventricular events	90 analyzable patients	NR	Novel MCG device (VitalScan)	Unshielded	Rotation Score, Angular Dynamics	Follow-up ICD therapy	Parameters associated with ICD therapy; exploratory AUC 0.82 and 0.77.	Prospective clinical	No
Ramirez et al., 2026 (103)	Fetal MCG signal quality	32 pregnant subjects (107 recordings)	OPM	15 OPMs (30 channels) bed-based array	3-layer CS	Mixed-effects regression	SNR and fetal CTI detectability	Higher gestational age increased SNR; shorter fetal-heart distance increased SNR.	Prospective clinical	No

acc., accuracy; AF, atrial fibrillation; AUC, area under curve; CAD, coronary artery disease; CAG, Coronary angiography; comp., comparison; CS, cylindrical shield; CTA, computed tomography angiography; CTI, cardiac time interval; ECG, electrocardiography; ECHO, echocardiography; FHR, fetal heart rate; ICA, independent component analysis; ICD, implantable cardioverter-defibrillator; IHD, ischemic heart disease; LAD, left anterior descending artery; LCX, left circumflex artery; LQTS, long QT syndrome; LVEF, left ventricular ejection fraction; MCG, magnetocardiography; MI, myocardial infarction; ML, machine learning; MSR, magnetically shielded room; NPV, negative predictive value; NR, not reported; NSTE-ACS, Non-ST-segment elevation acute coronary syndrome; NSTEMI, non-ST-segment elevation myocardial infarction; OPM, optically pumped magnetometer; PCD, pseudo current density; PVC, premature ventricular contraction; QTc, corrected QT interval; RCA, right coronary artery; SCD, sudden cardiac death; sens., sensitivity; SNR, signal-to-noise ratio; spec., specificity; SQUID, superconducting quantum interference device; SVM, support vector machine; TdP, Torsades de Pointes; tech., technology; UAP, unstable angina pectoris.

Studies are summarized for contextualization of bibliometric findings.

${}^{a}$The system was explicitly described as using SQUID sensors in a subsequent clinical study (109).

In the context of CAD, early clinical evidence was largely generated using SQUID-based MCG. For instance, Lim et al. demonstrated that a 64-channel shielded SQUID-MCG system could effectively detect non-ST-segment elevation myocardial infarction with high sensitivity (66). Subsequent studies demonstrated that SQUID-based MCG could also be acquired without a conventional magnetically shielded room. For example, Tao et al. used an unshielded SQUID-based system and machine learning models to detect ischemic heart disease and localize coronary stenosis (38). This illustrates that both acquisition environment and analytical strategy can evolve independently of the underlying sensor technology. More recently, OPM-based MCG has also entered adult CAD research. Han et al. combined spatiotemporal MCG features with machine learning methods using a multichannel OPM system to assess CAD severity and localize coronary stenosis (32). This provides technology-specific clinical evidence for OPM-MCG. In parallel, AI-driven analytical methods have advanced for multiple MCG systems. Bai et al. deployed a deep learning framework on OPM-MCG data to differentiate cardiac from non-cardiac chest pain (99). Meanwhile, Kranz et al. reported self-supervised representation learning based on 64-channel SQUID-MCG from a large cohort of 1,732 consecutive patients (63). Collectively, these studies support the growing analytical potential of AI-MCG, for both OPM-MCG and SQUID-MCG modalities.

In the realm of arrhythmology, MCG has been valued for its high spatial resolution. Early work by Fenici and Melillo (100) explored the use of MCG for the three-dimensional localization of arrhythmogenic substrates, suggesting its potential to assist in guiding invasive electrophysiology and ablation procedures. More recently, MCG-derived spatial and dynamic parameters have also been investigated under reduced-infrastructure acquisition conditions. In a prospective multicenter observational study, Lachlan et al. evaluated rotation score and angular dynamics using a portable, room-temperature MCG device without a magnetically shielded environment (101). Higher values were associated with subsequent appropriate ICD therapies, particularly shocks. While this study was small and exploratory, the 2026 report does not specify its underlying sensor technology. Accordingly, these findings primarily support the feasibility of portable and unshielded clinical MCG.

Fetal MCG serves a distinct clinical role in assessing prenatal arrhythmias and ANS development. Conventional ECG cannot reliably record fetal cardiac activity, as fetal electrical signals are severely attenuated by maternal abdominal volume conduction and the insulating vernix caseosa (102). Early multicenter studies established reference ranges for fetal cardiac time intervals using conventional MCG systems (69). More recently, OPM-based approaches have been explored for fetal MCG. Alem et al. demonstrated fetal MCG using a flexible array of microfabricated OPMs within a magnetically shielded room (59), illustrating that cryogen-free OPM sensors are capable of reliable fetal MCG recording. Importantly, Strand et al. directly compared SQUID- and OPM-based fetal MCG and found comparable signal-to-noise characteristics under their respective shielded acquisition configurations (71). This represents one of the relatively limited direct technology comparisons currently available. More recent OPM studies have extended fetal MCG toward clinically relevant measurements of fetal cardiac intervals and signal quality (103). Collectively, these studies illustrate the feasibility and expanding scope of OPM-based fetal MCG.

As MCG transitions from fundamental physiological research toward large-scale, real-world clinical applications, several critical challenges emerge. The most important translational challenge is therefore not simply technological advancement, but standardization across the entire MCG acquisition and interpretation pipeline (81). This includes sensor configuration, magnetic environment, field compensation, acquisition geometry, signal preprocessing, feature extraction, and clinical interpretation. Reporting these components explicitly is essential for determining whether apparently similar clinical findings are genuinely comparable across studies. Furthermore, it is essential to establish open-access, multicenter MCG databases similar to existing ECG repositories like PhysioBank (104).

Similarly, the rapid expansion of AI-based MCG analysis introduces a separate set of challenges. The lack of standardized acquisition protocols and open datasets limits model generalizability, while small single-center datasets increase the risk of overfitting. Explainability and external validation will therefore be essential for translating AI-based MCG models into clinical practice. Stringent regulatory oversight and standardized reporting frameworks are required to ensure these explainable AI models are formally integrated into global cardiovascular guidelines (105, 106).

### 4.4 Strengths, limitations and future directions

A key strength of this study lies in its dual-database approach (WoSCC and Scopus) and the integration of multiple bibliometric tools (VOSviewer, CiteSpace, R) to provide the first comprehensive, quantitative overview of the MCG field. However, several limitations must be acknowledged. First, the data sources were restricted to English-language articles and review articles. This approach inherently excludes gray literature, conference proceedings, and commercial patents. Because cutting-edge technical breakthroughs in MCG (such as OPM miniaturization, high-density array optimization, and active magnetic compensation) are often first disseminated through patents or engineering conference papers, relying solely on journal articles may cause a temporal lag. This limitation may impair precise timeline mapping of recent technological developments. Second, the restriction to English-language publications introduces a potential language bias, omitting high-quality research published by scholars in other languages. Third, bibliometric methodology inherently relies on quantitative indicators, such as citation counts and keyword frequencies, to assess academic impact. This approach is susceptible to publication bias and time-lag effects, wherein the academic impact of very recently published studies may be underestimated. More importantly, these quantitative metrics restrict the ability to conduct a thorough qualitative evaluation of the methodological rigor, sample representativeness, and conclusion reliability of the original studies (10, 107). In addition, bibliometric co-occurrence and citation patterns cannot determine whether a technological development has improved clinical performance, nor can they establish equivalence between different sensor platforms. The technology-stratified contextual synthesis presented in this study therefore serves to clarify the heterogeneity of the evidence base rather than to provide a formal comparative effectiveness assessment. Finally, the inherent time-lag in database indexing may lead to a temporary underrepresentation of the very latest technological breakthroughs.

Based on the evolutionary trajectory analyzed, future research in the MCG field is expected to advance in the following directions: (1) sensor technology innovation: efforts may focus on balancing the competing requirements of magnetic measurement. Future sensor development should seek to jointly optimize sensitivity, dynamic range, portability, environmental-field tolerance, and array scalability. Depending on the sensor architecture, this may involve OPM, TMR, NV, or improved SQUID-based systems, together with advances in passive shielding, active field compensation, and reference-sensor-based noise suppression. (2) comprehensive pipeline standardization: establishing consensus-based, standardized protocols covering the entire MCG workflow is crucial to eliminate methodological heterogeneity. These protocols should encompass hardware setup, signal acquisition, preprocessing, segmentation, and clinical data analysis (81). (3) explainable AI integration: as AI becomes deeply embedded in MCG diagnostics, future research must prioritize model transparency and explainability to meet the stringent safety and trust requirements of clinical medicine (108). (4) multicenter clinical trials: for mature diagnostic indications like CAD, standardized, international multicenter clinical trials are essential to accelerate the real-world clinical implementation of MCG. (5) novel application scenarios: continuous exploration of new cardiovascular application scenarios, such as routine sudden cardiac death risk stratification, may further maximize its clinical utility. (6) specific features identification: identifying highly sensitive and specific MCG features is necessary to improve diagnostic precision. Future studies can deeply explore the exact pathophysiological mechanisms underlying distinct spatiotemporal magnetic variations, establishing clear clinical correlates that link specific MCG signatures directly to distinct cardiac pathologies.

## 5 Conclusion

Since 2000, cardiovascular-related MCG research has evolved through the parallel development of sensing technologies, acquisition strategies, signal-processing methods, and clinical applications. Bibliometric evidence demonstrates that OPM-based instrumentation has become an increasingly prominent research direction, while SQUID-based systems continue to contribute substantially to clinical and translational studies. In parallel, AI-based methods, including machine learning and deep learning, have emerged as an independent and rapidly expanding analytical direction. Moving forward, future research should prioritize reduced-infrastructure sensor arrays, comprehensive standardization across the entire workflow, and explainable AI models. Overcoming these barriers will allow MCG to become an important complementary modality for ischemic heart disease detection, arrhythmia mapping, and fetal monitoring.

## Data Availability

The original contributions presented in the study are included in the article/**Supplementary Material**, further inquiries can be directed to the corresponding author.
